# Supplementary material for: Response of soil viral communities to land use changes
Source: Nat Commun. 2022 Oct 12;13:6027. doi: 10.1038/s41467-022-33771-2 (PMC9556555; doi:10.1038/s41467-022-33771-2)
Supplement: Supplementary file 1 — Supplementary Information file [file 41467_2022_33771_MOESM1_ESM.pdf]

Supplementary materials

Sampling site overview

Locations of sampling sites are indicated in Supplementary Figure 1, Sampling sites represented various types of land use with different locus were selected as follows. The extracellular viromes were named using sample name and intracellular viromes were named with an additional letter C before sample name. The major environmental factors across in soil samples were showed in Supplementary Data 1 as well.

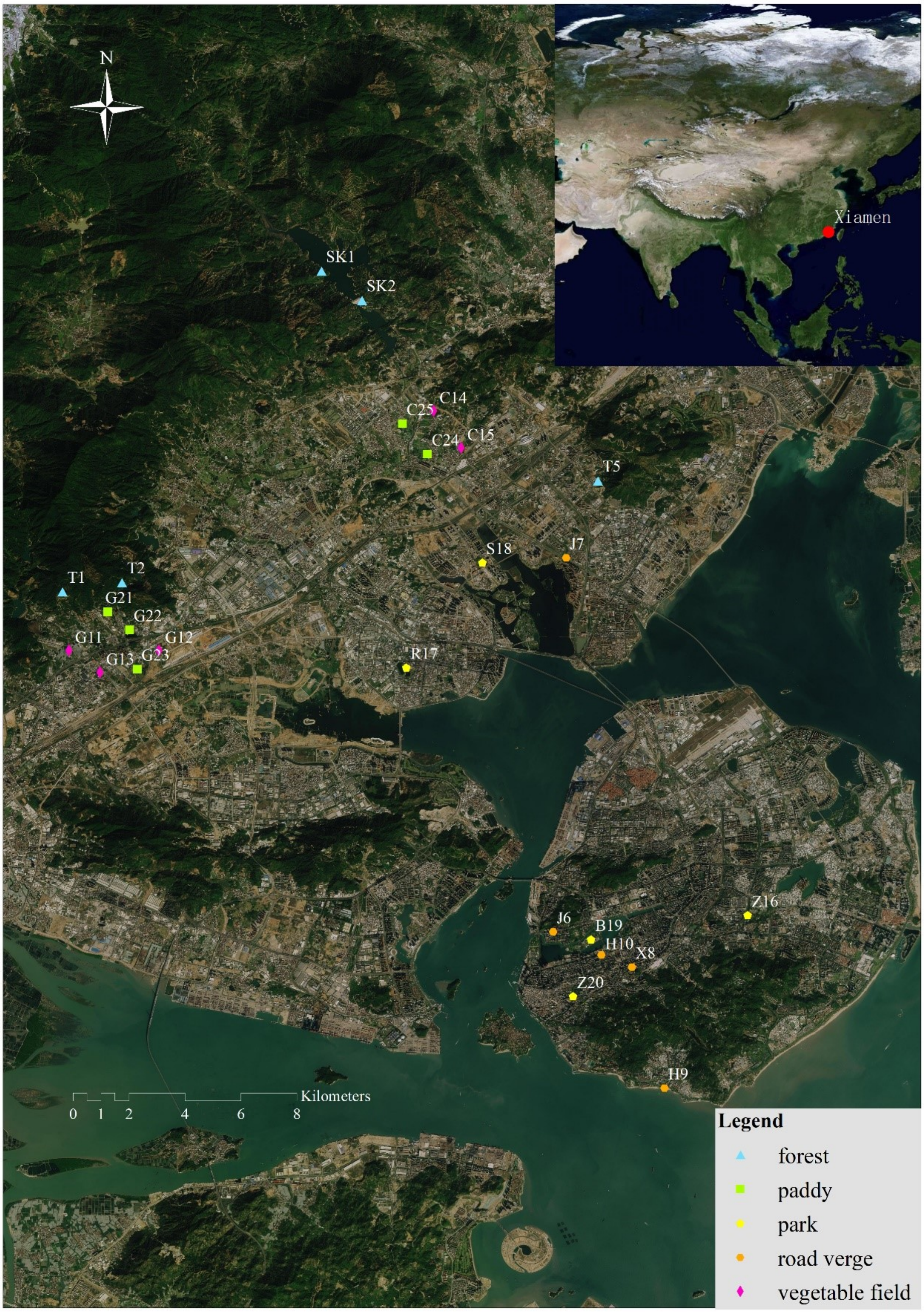

Supplementary Figure 1. Geographic location of sampling sites with five land-use types in Xiamen. Forest, T1 (Tianzhu mountain), T2 (Tianzhu mountain), SK1 (Tiejianshi mountain), SK2 (Tiejianshi mountain), T5 (Tianma mountain),

Road verge, J6 (Jimei avenue), J7 (Jianye Road), X8 (Xiahe Road), H9 (Huandao Road), H10 (Lakeside middle Road),

Vegetable field, G11 (Guankou town), G12 (Guankou town), G13 (Guankou town), C14 (Chaichang), C15 (Chaichang),

Park, Z16 (Zhonglun park), R17 (Ridong park), S18 (Shiming park), B19 (Bailuzhou park), Z20 (Zhongshan park)

Paddy, G21 (Guankou town), G22 (Guankou town), G23 (Guankou town), C24 (Chaichang), C25 (Chaichang)

The base map of the satellite reprinted with permission from website http://lbs.tianditu.gov.cn/server/MapService.html.

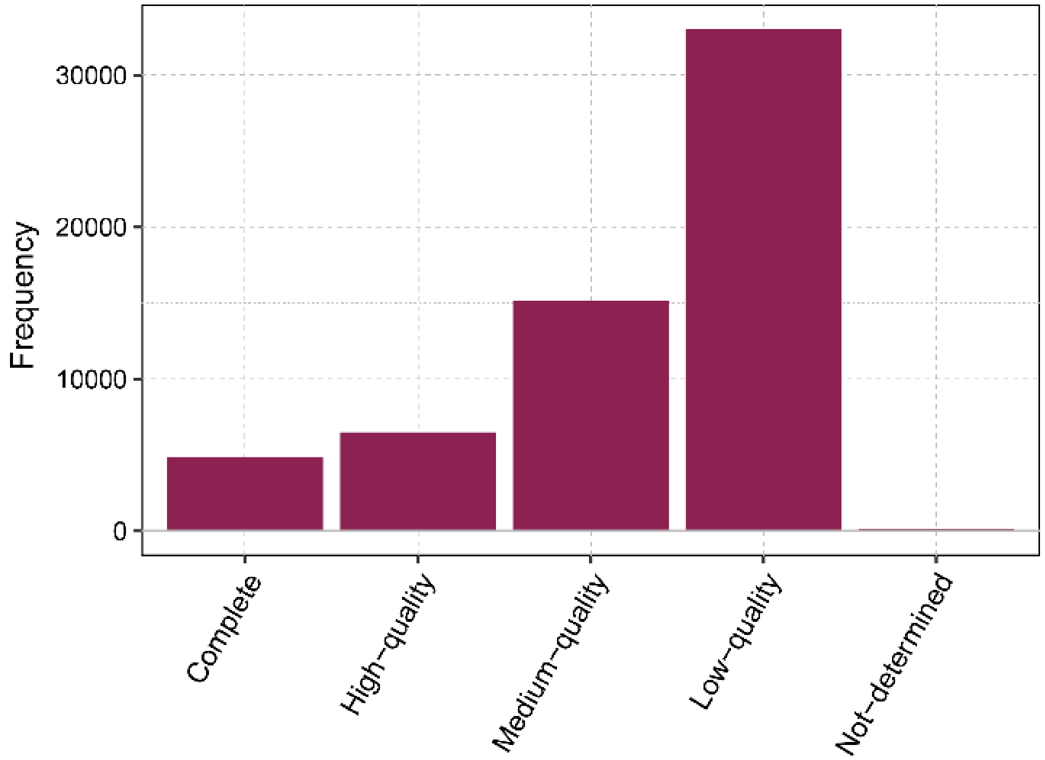

Supplementary Figure 2. Quality assessment of 59,626 vOTUs by checkV analysis.

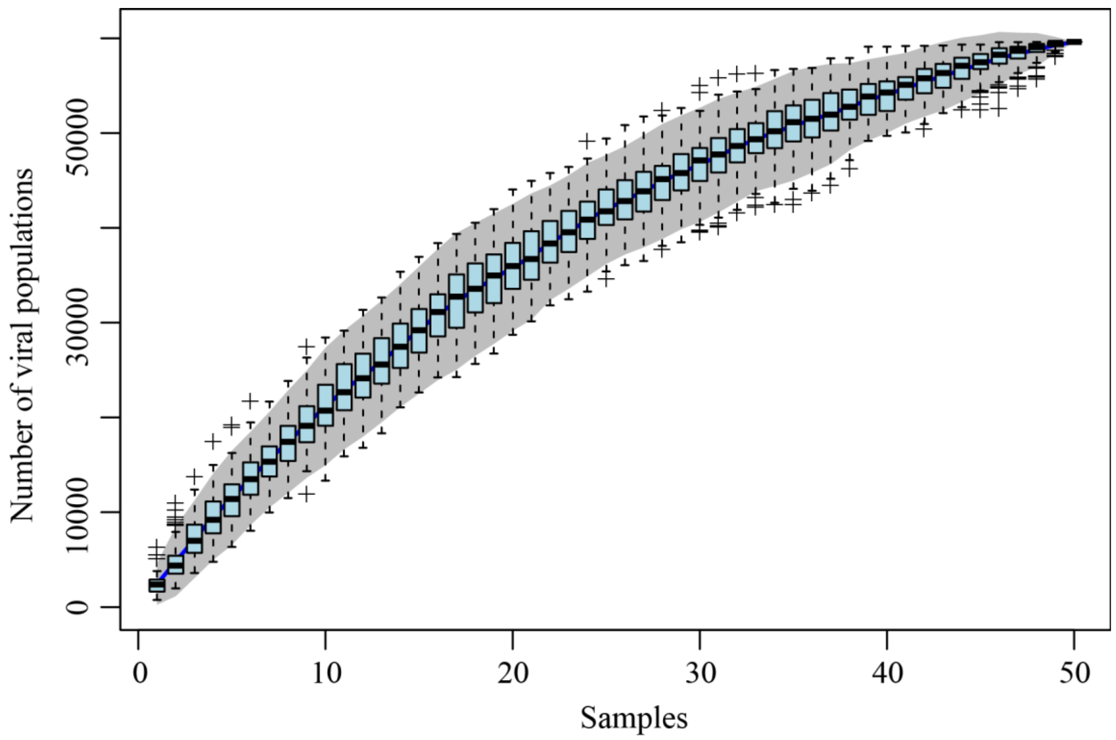

Supplementary Figure 3. Accumulative curve of vOTUs detected from 50 viromes subsampled to 30M reads. The minima, maxima, centre, bounds of box and whiskers in boxplots from bottom to top represented percentile 0, 10, 25, 50, 75, 90 and 100, respectively.

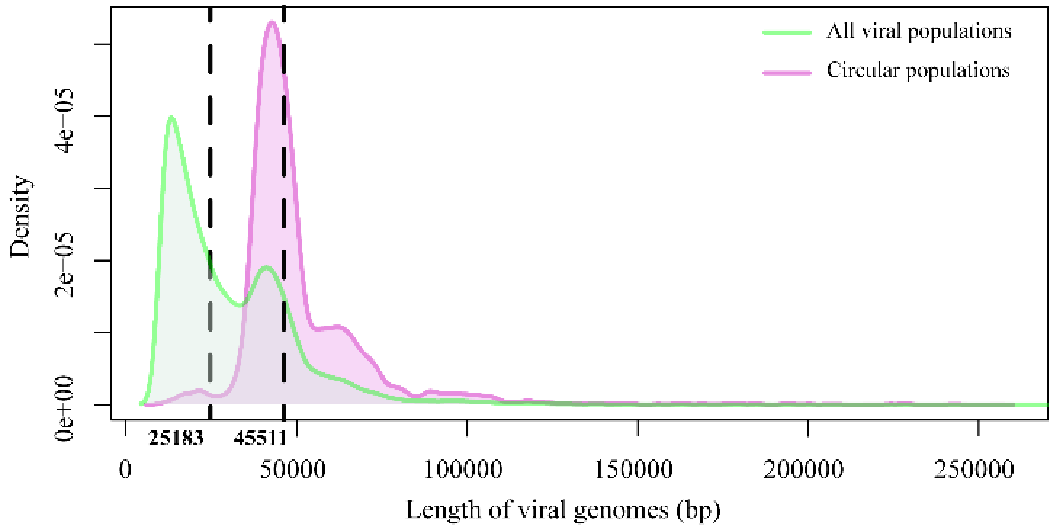

Supplementary Figure 4. Genomic length distribution of the viral dataset including 59,626 VPs (blue line) and 8,112 VPs with completed circular genomes (purple line) identified by VIBRANT.

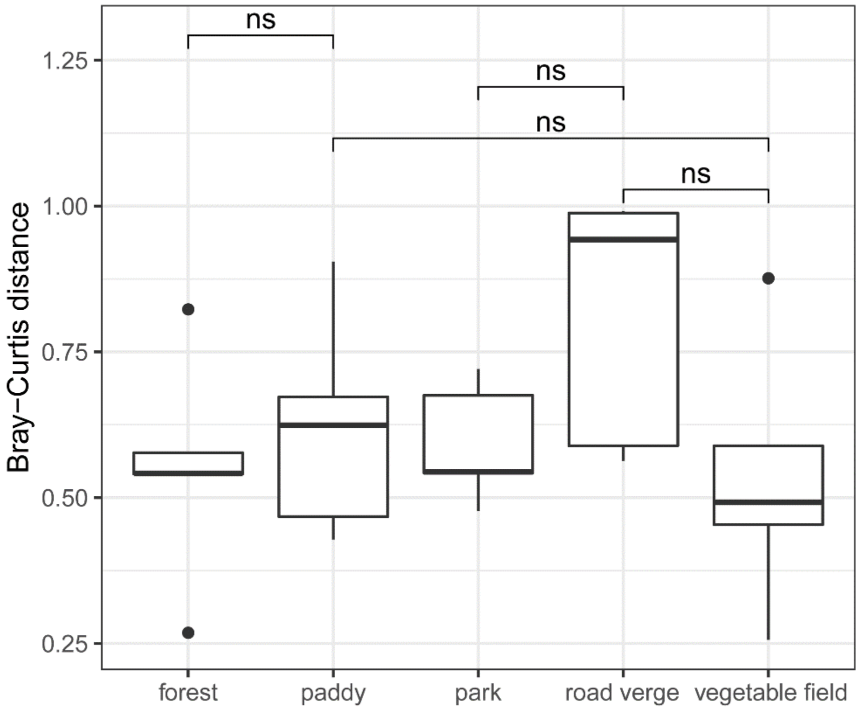

Supplementary Figure 5. Boxplot showing the Bray-Curtis distance between paired intracellular and extracellular viral communities in different land uses (n=5). The minima, maxima, centre, bounds of box and whiskers in boxplots from bottom to top

represented percentile 0, 10, 25, 50, 75, 90 and 100, respectively, ns represents $p >$ 0.05. The statistical test used was two-tailed.

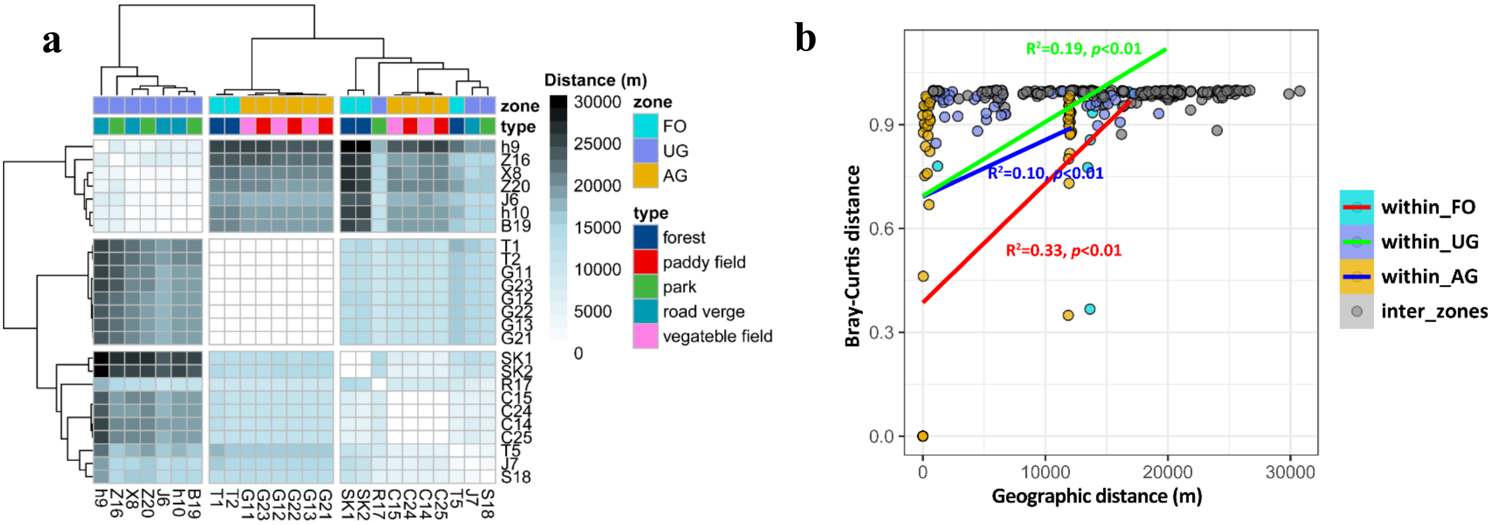

Supplementary Figure 6. (a) Heatmap showed the clustering of spatial distance (m) between different viral communities. The land use zone AG represents agricultural areas including paddy and vegetable field; UG represents urban green space including park and road verge; FO represents forest. (b) Geographic decay effect of viral communities within different zones. Inset values display the R^2 and adjusted p value of F-statistic. The statistical test used was two-tailed.

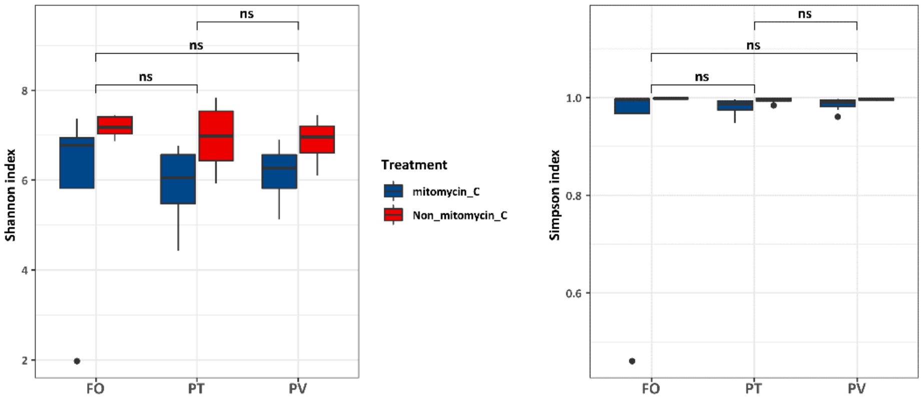

Supplementary Figure 7. The alpha diversity Shannon (left) and Simpson (right) index of viral communities across different zones with different treatments. The land use zone AG (n=5) represents agricultural areas including paddy and vegetable field; UG (n=10) represents urban green space including park and road verge; FO (n=10) represents forest. The minima, maxima, centre, bounds of box and whiskers in boxplots from bottom to top represented percentile 0, 10, 25, 50, 75, 90 and 100, respectively, the difference between different zones was tested using the Wilcox.test, ns represents $p > 0.05$.

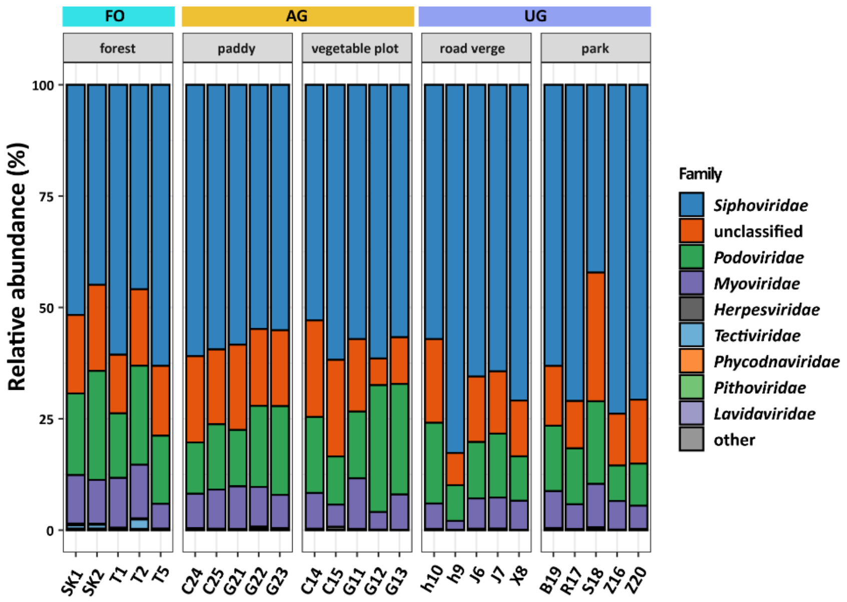

Supplementary Figure 8. Family distribution of soil viral community compositions. The land use zone AG represents agricultural areas including paddy and vegetable field; UG represents urban green space including park and road verge; FO represents forest.

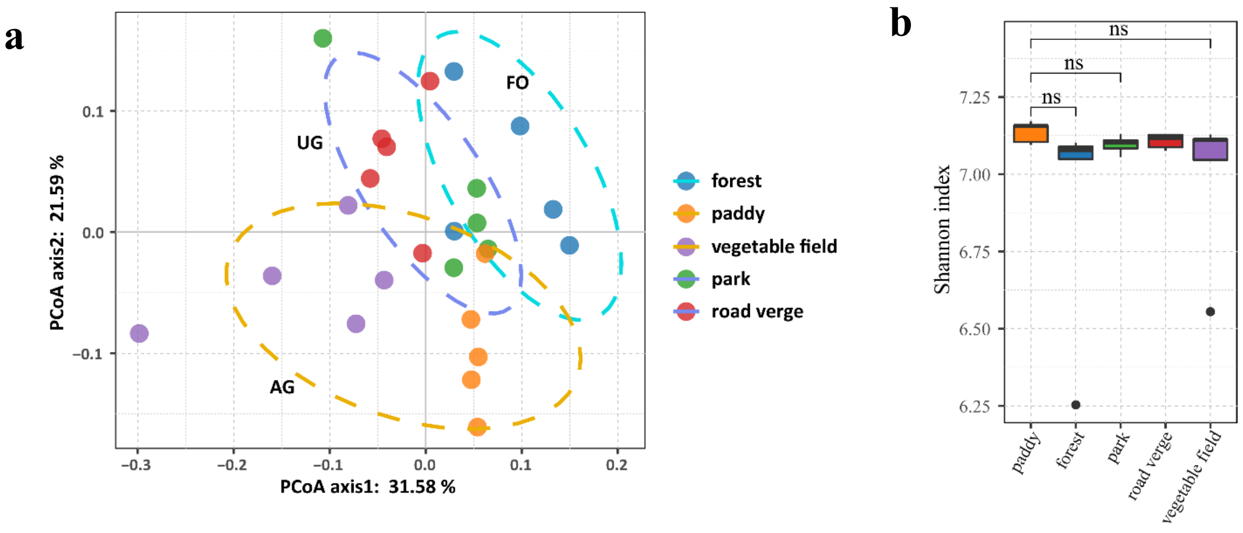

Supplementary Figure 9. (a) Principal coordinates analysis (PCoA) of microbial community structures. The analysis of similarity (ANOSIM) was used to test the significance of difference among land use types. The land use zone AG (n=5) represents agricultural areas including paddy and vegetable field; UG (n=10) represents urban green space including park and road verge; FO (n=10) represents forest. Ellipses in the PCoA plot are drawn around the centroids of each zone at 80% confidence intervals. (b) Shannon index of microbial community structures for each land use (n=5). The minima, maxima, centre, bounds of box and whiskers in boxplots from bottom to top represented percentile 0, 10, 25, 50, 75, 90 and 100, respectively. All pairwise comparisons shown were performed using two-sided Wilcox.test. p values lower than 0.05 were denoted as ‘‘ns.’’

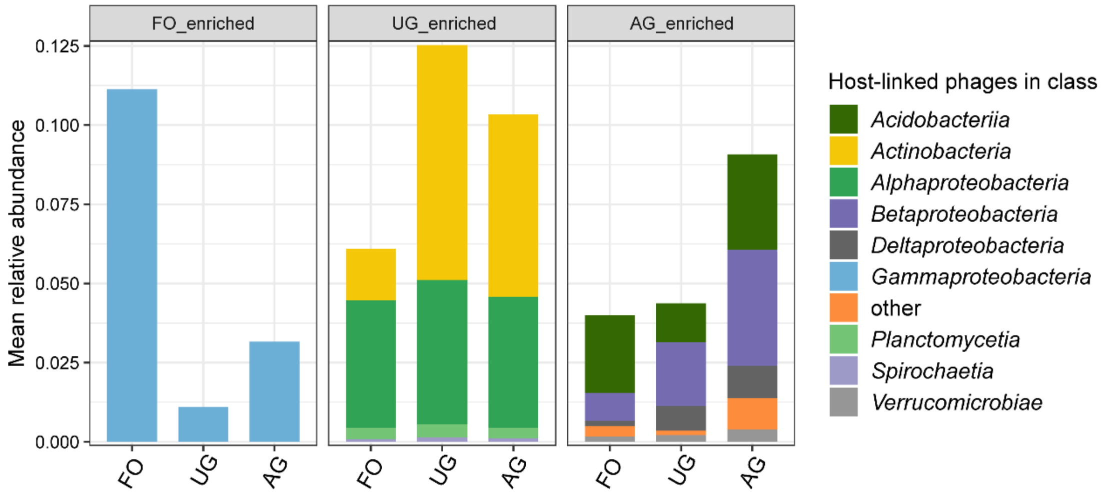

Supplementary Figure 10. Shifts in the mean summed host-linked relative abundances of significantly FO-enriched, UG-enriched, and AG-enriched VPs in class-level across the three zones. The land use zone AG represents agricultural areas including paddy and vegetable field; UG represents urban green space including park and road verge; FO represents forest.

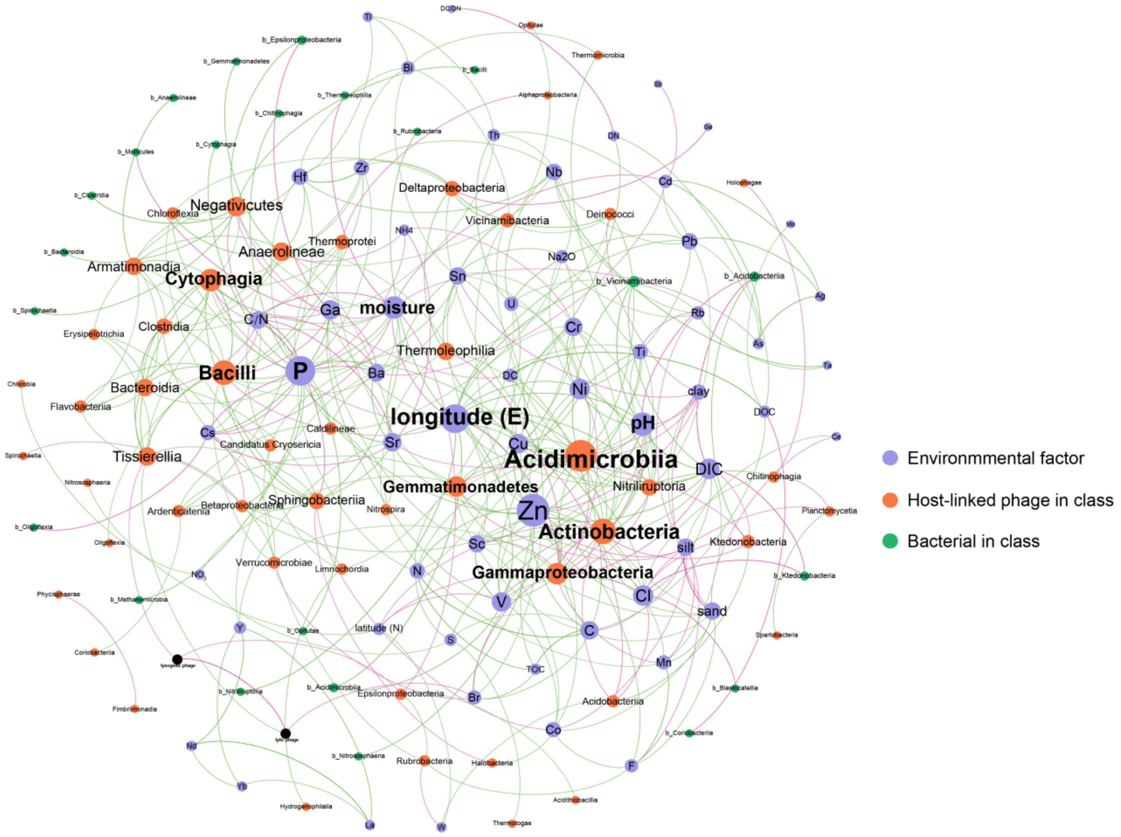

Supplementary Figure 11. Network analysis depicting an interaction pattern among host-linked vOTUs in class, bacteria in class and environmental factor. The size of nodes and labels represents the degree of connection. The layout method of this network was Fruchterman Reingold. The blue line indicated the positive interaction and red line indicated negative interaction.

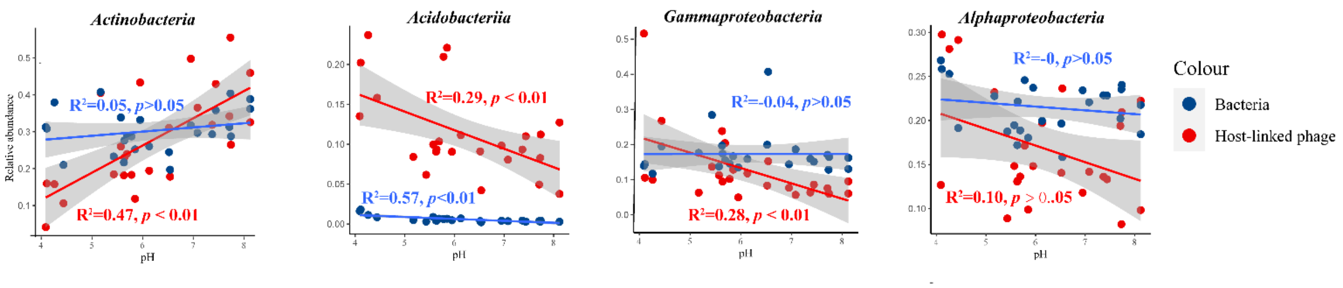

Supplementary Figure 12. The linear model showing the relationship between the relative abundance of host-linked phage (red line) and bacteria (blue line) and pH. The fitted line is provided for 95% confidence intervals. Inset values display the R^2 and adjusted *p* value of F-statistic. The statistical test used was two-tailed. Source data are provided in the Source Data file.

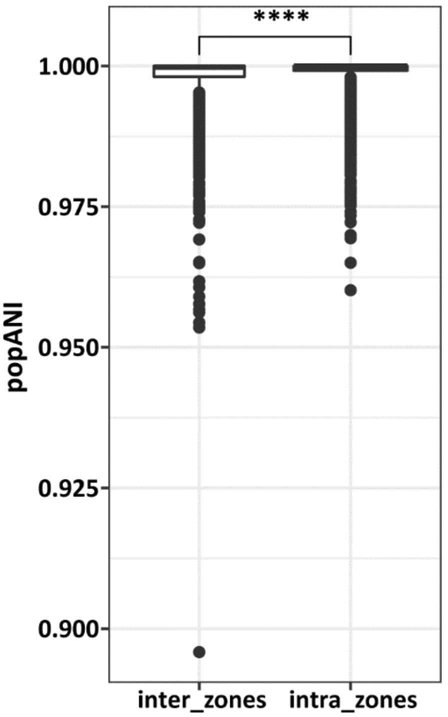

Supplementary Figure 13. The boxplot indicated that the multi-zonal vOTUs have a significant bigger popANI intra zones (n=5,658) compare with inter-zones (n=3,606). The minima, maxima, centre, bounds of box and whiskers in boxplots from bottom to top represented percentile 0, 10, 25, 50, 75, 90 and 100, respectively. The *p* value from t.test; **** represents $p<0.0001$. The exact *p* value less than 2.2e-16. The statistical test used was two-tailed.
