# Supplementary material for: Response of soil viral communities to land use changes
Source: Nat Commun. 2022 Oct 12;13:6027. doi: 10.1038/s41467-022-33771-2 (PMC9556555; doi:10.1038/s41467-022-33771-2)
Supplement: Supplementary file 2 — Reporting Summary [file 41467_2022_33771_MOESM2_ESM.pdf]

nature portfolio

Corresponding author(s): Jian-Qiang Su

Last updated by author(s): May 10, 2022

Reporting Summary

Nature Portfolio wishes to improve the reproducibility of the work that we publish. This form provides structure for consistency and transparency in reporting. For further information on Nature Portfolio policies, see our Editorial Policies and the Editorial Policy Checklist.

Statistics

For all statistical analyses, confirm that the following items are present in the figure legend, table legend, main text, or Methods section.

n/a	Confirmed	
☐	☒	The exact sample size (n) for each experimental group/condition, given as a discrete number and unit of measurement
☐	☒	A statement on whether measurements were taken from distinct samples or whether the same sample was measured repeatedly
☐	☒	The statistical test(s) used AND whether they are one- or two-sided<br>*Only common tests should be described solely by name; describe more complex techniques in the Methods section.*
☐	☒	A description of all covariates tested
☐	☒	A description of any assumptions or corrections, such as tests of normality and adjustment for multiple comparisons
☐	☒	A full description of the statistical parameters including central tendency (e.g. means) or other basic estimates (e.g. regression coefficient) AND variation (e.g. standard deviation) or associated estimates of uncertainty (e.g. confidence intervals)
☐	☒	For null hypothesis testing, the test statistic (e.g. F, t, r) with confidence intervals, effect sizes, degrees of freedom and P value noted<br>*Give P values as exact values whenever suitable.*
☒	☐	For Bayesian analysis, information on the choice of priors and Markov chain Monte Carlo settings
☒	☐	For hierarchical and complex designs, identification of the appropriate level for tests and full reporting of outcomes
☐	☒	Estimates of effect sizes (e.g. Cohen's d, Pearson's r), indicating how they were calculated

Our web collection on statistics for biologists contains articles on many of the points above.

Software and code

Policy information about availability of computer code

Data collection	We used our basic data

Data analysis	cutadpat v2.11<br>trimmomatic v0.39<br>The custom code have been deposited in https://github.com/liaohu1231/Virome.git (https://doi.org/10.5281/zenodo.7041414)<br>Kraken2 v2.0.7-beta<br>Bracken<br>vegan<br>metaspades v3.13.0<br>VIBRANT v1.2.1<br>HMMER 3.3.2<br>Cluster_genomes_5.1.pl (https://github.com/simroux/ClusterGenomes)<br>CheckV v0.7.0<br>vConTACT2<br>Demovir script (https://github.com/feargalr/Demovir)<br>VirHostMatcher-Net<br>Seqtk v1.3<br>CoverM v0.5.0<br>bowtie2<br>inStrain v1.5.3<br>CD-HIT v4.6<br>InterProScan v5

For manuscripts utilizing custom algorithms or software that are central to the research but not yet described in published literature, software must be made available to editors and reviewers. We strongly encourage code deposition in a community repository (e.g. GitHub). See the Nature Portfolio guidelines for submitting code & software for further information.

Data

Policy information about availability of data

All manuscripts must include a data availability statement. This statement should provide the following information, where applicable:

- Accession codes, unique identifiers, or web links for publicly available datasets
- A description of any restrictions on data availability
- For clinical datasets or third party data, please ensure that the statement adheres to our policy

The raw reads from Illumina viromes sequencing have been deposited in the NCBI under the project PRJNA691683. Raw reads from Illumina metagenomes sequencing were submitted to the NCBI under the project PRJNA746419. Clean reads from Illumina viromes sequencing were submitted to the ScienceDB (https://www.scidb.cn/s/yiIfAv). The database LVD have been deposited in figshare (10.6084/m9.figshare.19108391). All bona-fide viral genomes of database LVD have been deposited in figshare (10.6084/m9.figshare.19740130). In additions, we also deposited another version of LVD which vOTUs clustered at thresholds of 95% average nucleotide identity over 85% alignment fraction using Perl script Cluster_genomes_5.1.pl into figshare (10.6084/m9.figshare.19743646). The TrEMBL database have been deposited in https://figshare.com/articles/NR_Viral_TrEMBL/5822166. Source data are provided with this paper.

Field-specific reporting

Please select the one below that is the best fit for your research. If you are not sure, read the appropriate sections before making your selection.

☐ Life sciences ☐ Behavioural & social sciences ☒ Ecological, evolutionary & environmental sciences

For a reference copy of the document with all sections, see nature.com/documents/nr-reporting-summary-flat.pdf

Ecological, evolutionary & environmental sciences study design

All studies must disclose on these points even when the disclosure is negative.

Study description	we conducted an in-depth characterization of soil viral composition and spatial distribution across five land use types including forest, paddy field, vegetable field, urban park, and road verge through viromic analysis. Host-linked interactions, lysogenicity, and microdiversity were further investigated to illustrate the ecological and evolutionary adaptation of soil virome to land use changes.
Research sample	Samples were collected from soil with various types of uses, including forest, paddy field and vegetable field, urban park and road verge in Xiamen, China on Jul-26 2020, transported to lab on ice bag in a foam box, and stored immediately in 4 oC, which were further grouped into three land use zones as forest zone (FO), agricultural zone (AG), and urban green space zone (UG). These soils represent the major land use types, and are closely related to anthropogenic activities. Each land use type consisted of five randomly selected locations, and five replicate topsoil (0-20 cm) samples were collected and pooled into a composite sample from each location, resulting in a total of 25 soil samples
Sampling strategy	These soils represent the major land use types, and are closely related to anthropogenic activities. Each land use type was consisted of five locations, and five replicate topsoil (0-20 cm) samples were collected from each location, resulting in a total of 25 soil samples.
Data collection	Soil viral DNA was extracted and sequenced. Physi-chemical properties of soil was measured in our lab. All the data were collected by the first author Hi Liao. All experments were recorded in Experimental Record Book

Timing and spatial scale	Start time:Jul-26 2020<br>End Time: Jul-26 2020<br>Spatial scale: Xiamen, China
Data exclusions	No data was excluded
Reproducibility	All replicate soil samples were successful for DNA extraction and sequencing one time.
Randomization	We randomly collected soil sample from 25 locations with five types of land use, each land use randomly collected five samples.
Blinding	The investigators were blinded to group allocation during data collection and/or analysis

Did the study involve field work? ☒ Yes ☐ No

Field work, collection and transport

Field conditions	Soil samples were colleted on Jul 2020 at sunny days. The temperature was 32-35 degree.
Location	Soil samples were collected in Xiamen, China. N24°23'～24°54', E117°53'～118°26', Elevation 5-40 m
Access & import/export	Sample collection was permitted by the Garden Bureau of Xiamen City.
Disturbance	Disturbance the soil surface in the sampling place weakly, but the excess soil sample was refilled to the sampling place.

Reporting for specific materials, systems and methods

We require information from authors about some types of materials, experimental systems and methods used in many studies. Here, indicate whether each material, system or method listed is relevant to your study. If you are not sure if a list item applies to your research, read the appropriate section before selecting a response.

Materials & experimental systems

n/a	Involved in the study	
☒	☐	Antibodies
☒	☐	Eukaryotic cell lines
☒	☐	Palaeontology and archaeology
☒	☐	Animals and other organisms
☒	☐	Human research participants
☒	☐	Clinical data
☒	☐	Dual use research of concern

Methods

n/a	Involved in the study	
☒	☐	ChIP-seq
☒	☐	Flow cytometry
☒	☐	MRI-based neuroimaging
