# Supplementary figures and images for: Response of soil viral communities to land use changes
Source: Nat Commun. 2022 Oct 12;13:6027. doi: 10.1038/s41467-022-33771-2 (PMC9556555; doi:10.1038/s41467-022-33771-2)

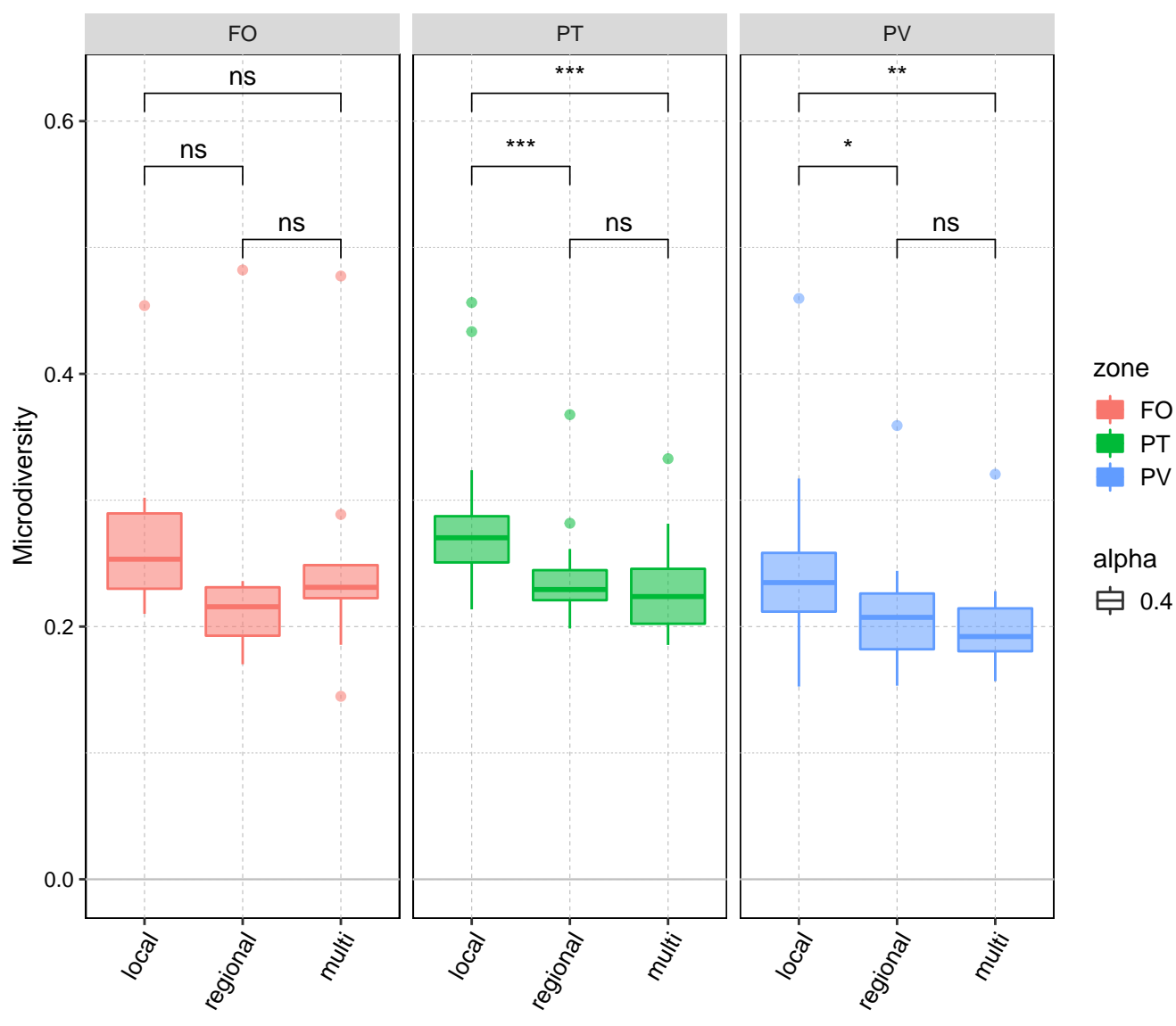

Supplement: Supplementary file 13 — source data [file 41467_2022_33771_MOESM13_ESM.zip › source_data/Figure 6_microdiverisity/pNpS_populations_zone.pdf]
